# Novel structure in the nuclei of honey bee brain neurons revealed by immunostaining

**DOI:** 10.1038/s41598-021-86078-5

**Published:** 2021-03-25

**Authors:** Paul J. Hurd, Kornelia Grübel, Marek Wojciechowski, Ryszard Maleszka, Wolfgang Rössler

**Affiliations:** 1 School of Biological and Chemical Sciences, Queen Mary University of London, London, E1 4NS UK; 2 Behavioral Physiology and Sociobiology (Zoology II), Biozentrum, University of Würzburg, Am Hubland, 97074 Würzburg, Germany; 3 Research School of Biology, The Australian National University, Canberra, ACT 2601 Australia

**Keywords:** Biological techniques, Cell biology, Developmental biology, Molecular biology, Neuroscience

## Abstract

In the course of a screen designed to produce antibodies (ABs) with affinity to proteins in the honey bee brain we found an interesting AB that detects a highly specific epitope predominantly in the nuclei of Kenyon cells (KCs). The observed staining pattern is unique, and its unfamiliarity indicates a novel previously unseen nuclear structure that does not colocalize with the cytoskeletal protein f-actin. A single rod-like assembly, 3.7–4.1 µm long, is present in each nucleus of KCs in adult brains of worker bees and drones with the strongest immuno-labelling found in foraging bees. In brains of young queens, the labelling is more sporadic, and the rod-like structure appears to be shorter (~ 2.1 µm). No immunostaining is detectable in worker larvae. In pupal stage 5 during a peak of brain development only some occasional staining was identified. Although the cellular function of this unexpected structure has not been determined, the unusual distinctiveness of the revealed pattern suggests an unknown and potentially important protein assembly. One possibility is that this nuclear assembly is part of the KCs plasticity underlying the brain maturation in adult honey bees. Because no labelling with this AB is detectable in brains of the fly *Drosophila melanogaster* and the ant *Camponotus floridanus*, we tentatively named this antibody AmBNSab (*Apis mellifera* Brain Neurons Specific antibody). Here we report our results to make them accessible to a broader community and invite further research to unravel the biological role of this curious nuclear structure in the honey bee central brain.

## Introduction

Since the publication of the honey bee draft genome in 2006^1,2^, there has been a significant shift in research priorities involving this highly social insect. In particular, a rapid development of molecular tools has facilitated all sorts of genomic, transcriptomics and epigenomic studies^3–6^. However, a limited availability of specific honey bee antibodies has been a critical factor hampering several lines of research that often require a precise localization of a given protein in cells or tissues, in particular those relevant to brain and behaviour. Given the prevalence of antibody usage in detecting specific molecules in vivo and in vitro^7^, this scarcity of antibodies is a potential disadvantage that reduces experimental testability in honey bees versus other invertebrate species like *D. melanogaster* or *C. elegans.* Although both the fly and the nematode are gold standards for many areas of biomedical sciences^8,9^, the honey bee with its elaborate social structure and diet-controlled phenotypic polymorphism has much to offer in the study of behaviour and development^10,11^. The three organismal outcomes derived from the same genotype, the two female castes and male drones, are strikingly different in their anatomy, longevity and respective behaviours, and their brains show remarkable structural plasticity during adult behavioural maturation^12–15^. In recent years, the honey bee emerged as a convenient system in which the interactions between gene regulatory networks involving DNA-binding proteins and the epigenomic decorations associated with DNA methylation can be analysed^16,17^. This important aspect of cellular regulation cannot be investigated in flies and nematodes that lost the DNA methylation enzymology and cannot provide meaningful input into the function of epigenomic communication systems^4,18^.

This project is part of our interest in identifying and characterising molecules controlling cellular processes in the brain and ultimately behaviour via a set of mechanisms broadly referred to as epigenetic^19^. An important aspect of this line of research is the development of antibodies to advance functional classification of proteins predicted to be relatives of the mammalian epigenetic toolkit. The honey bee proteome is mostly annotated by comparative analyses using datasets of model species. However, many important differences between various lineages suggest that functional transferability based exclusively on sequence comparisons is not sufficient to imply species-specific roles^10,20,21^. It is now clear that data transfer between the genome/epigenome, transcriptome and phenome needs to include the co- and post-translational levels and antibody-based molecular anatomy^22^. Here we report a characterisation of a newly produced antibody AmBNSab that detects an unusual nuclear structure predominantly in the intrinsic neurons (Kenyon cells) of the honey bee mushroom bodies (MBs), a prominent bilateral neuropil found in the brains of most arthropods^23^. Some features of the observed immunostaining pattern suggest that this nuclear structure is not only novel but might be specific to the honey bee.

## Results and discussion

Our initial aim was to generate an antibody against the single honey bee relative of mammalian ten-eleven translocation methylcytosine dioxygenases (TETs)^24^, a family of proteins implicated in numerous cellular activities including DNA demethylation and cooperative regulation of gene networks^25^. As it often happens, an antibody produced to bind to one protein can bind to another^26^, and as shown in this case, with excellent affinity. The result of a Western blot shown in figure S1A indicates that this new AB detects a large > 300 kD protein in the brain extract of adult honey bee foragers. Only a single and clear band is visible suggesting that the antibody has a high level of specificity for only one protein expressed in the brain. However, the antibody failed to detect the target epitope in the context of a HA-tagged AmTet_Cat_ construct after transfection into human embryonic kidney (HEK) 293 cells (Figure S1B). Given this result, the antibody was deemed to be novel, and subsequently named AmBNSab (see below). Following the initial validation, we performed high-resolution microscopy to examine the intracellular localisation of AmBNSab. We used adult brains dissected from two female castes, workers, and queens, as well as from males (drones). The drones have quite unusual brain anatomy dominated by huge optic lobes with the mushroom bodies somewhat hidden underneath. In addition, we examined the AmBNSab labelling pattern in preadult stages, namely larvae of various ages, and stage 5 pupae^27^. The aim of this experiment is to determine if the new nuclear structure is present before and through metamorphosis, during which the nervous system is reorganised to replace most larval neurons with adult neurons^28^.

**Figure 1:**
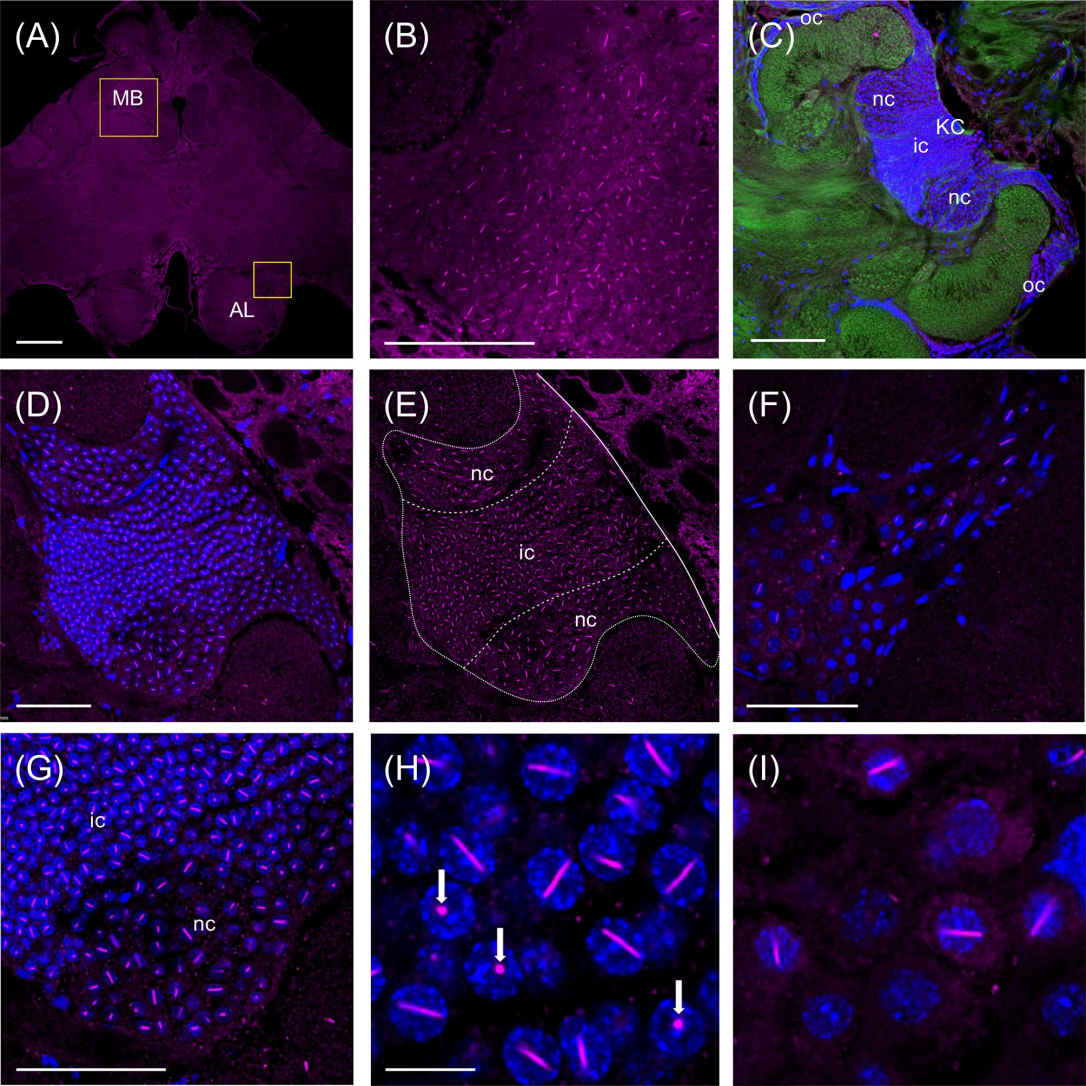
Immunolocalization of AmBNSab in the brain of adult honey bee workers. (**A**) Overview of the honey bee brain labeled with AmBNSab (magenta) at low magnification shows evenly distributed low background in most parts of the brain. (**B**) At higher magnification AmBNSab specific labeling in the mushroom body (MB) calyx (area defined by the box in a) reveals rod-like assemblies in KC nuclei. (**C**) Detail of one MB calyx double labeled with Hoechst 34580 (blue) and f-actin phalloidin (green) as orientation for the following figures. The localization of inner compact (ic), inner non-compact (nc) and outer compact (oc) Kenyon cells (KC) is indicated. (**D**,**E**) Distinct labeling with AmBNSab in KC nuclei in the MB calyx cup. Double labeling with Hoechst 34580 (blue) and AmBNSab (magenta) in (**D**) and AmBNSab channel only (magenta) in (**E**). The lines in (**E**) demarcate the border between the KC nuclei belonging to the inner compact and non-compact KC nuclei. (**G,H**) Double stained KC nuclei at higher magnification. Labeling with anti-AmBNSab (magenta) and cell nuclei staining with Hoechst 34580 (blue). Overview of the inner compact and non-compact (**G**) and high magnification of non-compact KC nuclei in (**H**). Both KC types contain AmBNSab positive rod-like structures with variable spatial orientation (**G**,**H**). The arrows in (**H**) indicate KC nuclei where the rod-like assemblies are viewed in a cross section. (**F**,**I**) In contrast to the MBs, the antennal lobe (AL) and other parts of the brain (not shown) contain no or much smaller numbers of cell nuclei with AmBNSab positive rod-like structures. Overview of cell nuclei in a cluster of AL neurons (**F**), shown at higher magnification in (**I)**. Only few somata contain cell nuclei with AmBNSab positive labeling. Scale bars: **A** = 200 µm, **B** = 50 µm, **C** = 100 µm, **D**,**E** = 50 µm, **F** = 50 µm, **G** = 50 µm, **H**–**I** = 10 µm. Five brains were analysed.

**Figure 2:**
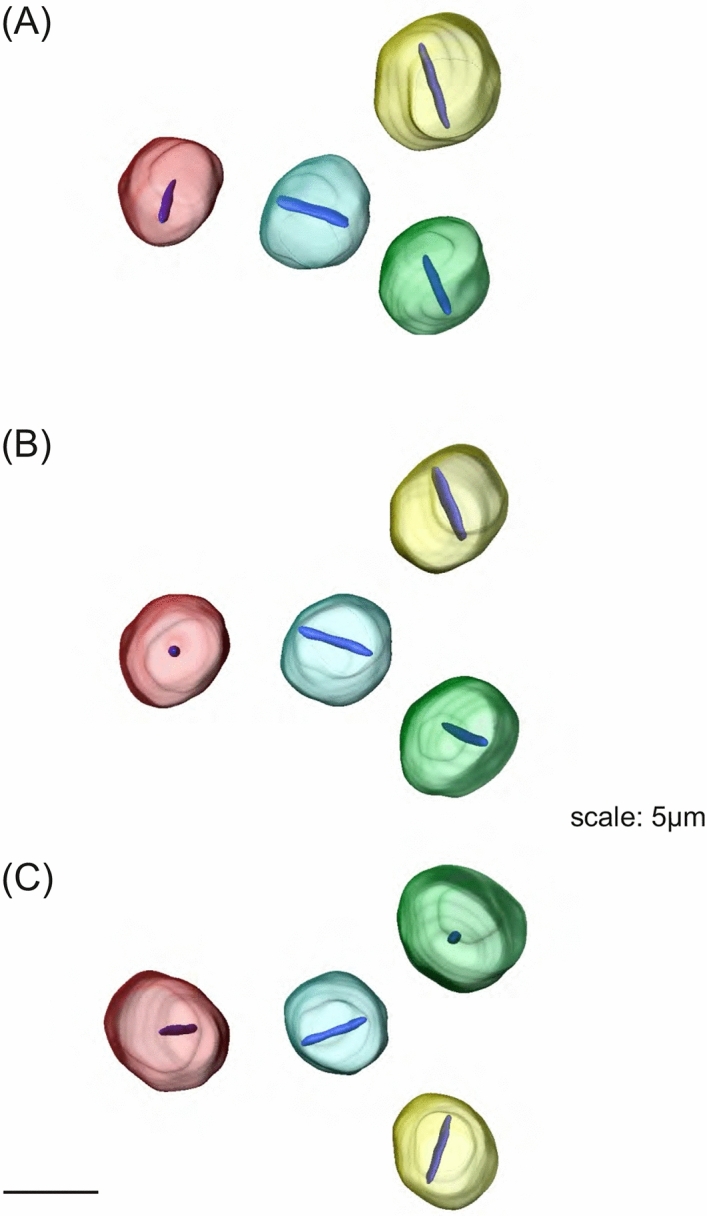
3D-reconstructions of individual Kenyon cell (KC) nuclei containing the rod-like assembly of AmBNSab labeling. (**A–C**) Random selection of 4 different KC nuclei from the group of inner non-compact KCs shown in different orientations. Each nucleus is shown in a different colour. The average length (N = 10) of the rod-like assemblies (blue) in this group of KCs is 4.1 µm. Scale bar = 5 µm.

**Figure 3:**
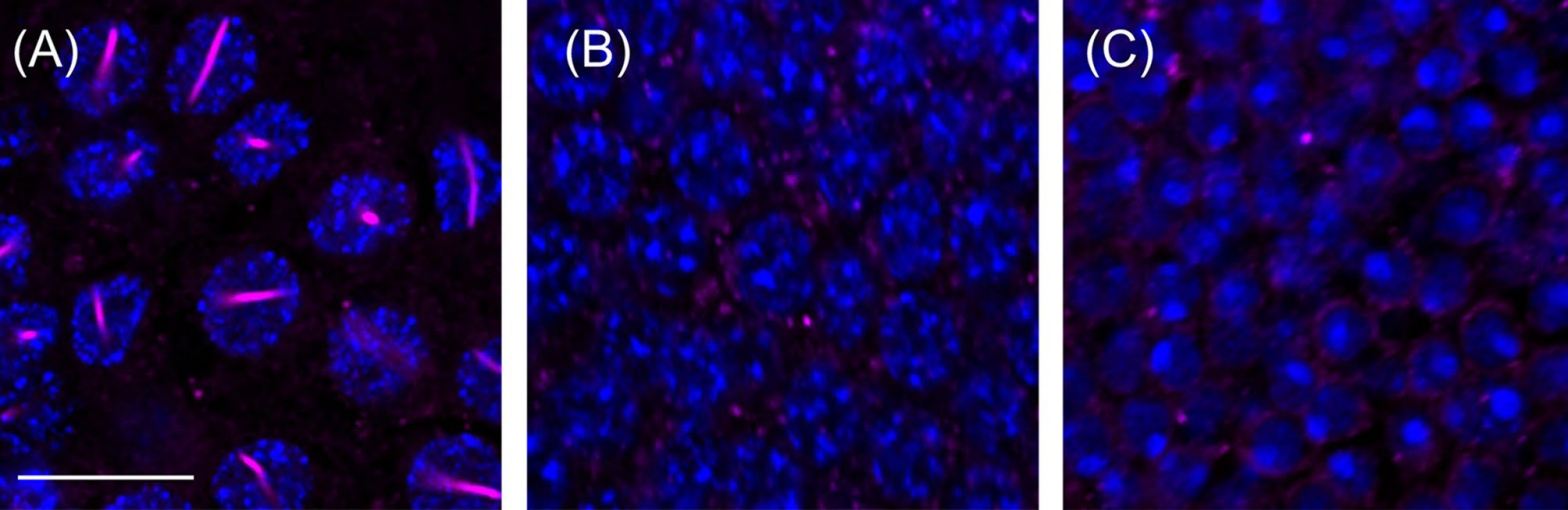
Labeling of rod-like assemblies in Kenyon cell (KC) nuclei with AmBNSab is specific in honey bee brains*.* (**A–C**) Comparison of labeling of KC nulcei in the mushroom body of honeybee (*Apis mellifera*) worker (**A**) with labeling in the carpenter ant, *Camponotus floridanus* (**B**) and the fruit fly, *Drosophila melanogaster* (**C**), all shown at the same magnification. KC nuclei labeled with Hoechst 34580 in blue and staining with AmBNSab in magenta. Rod-like structures are present only in KC nuclei of the honey bee brain. Scale bar in a, also valid for **B**,**C** = 10 µm. Number of brains analysed: **A**.* mellifera* = 2, **C**.* floridanus* = 4; **D**.* melanogaster* = 4.

**Figure 4:**
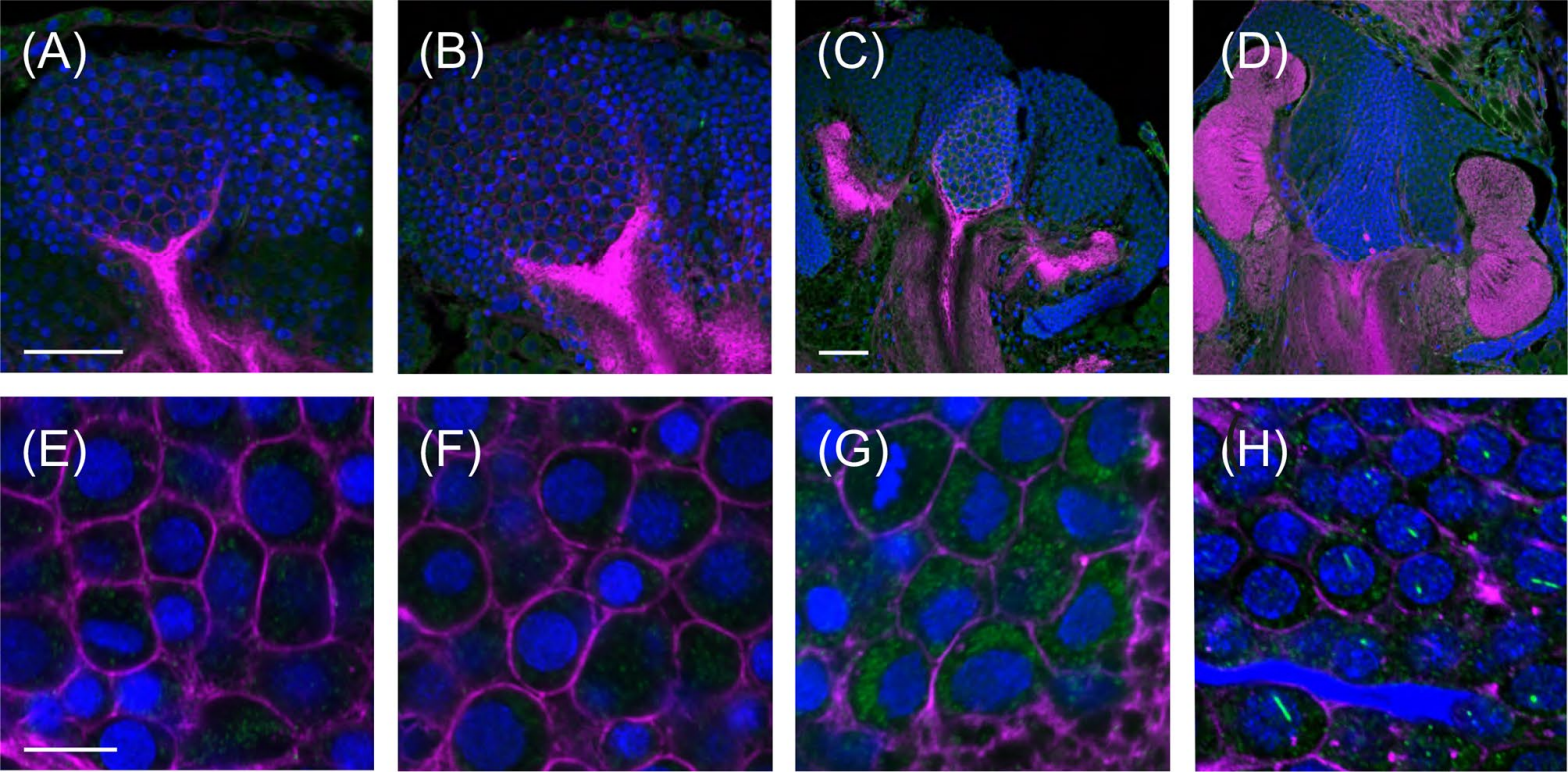
(**A–H**) Life-stage specific differences in labeling of Kenyon cell (KC) nuclei with AmBNSab in honey bees. Staining of cell nuclei with Hoechst 34580 (blue), f-actin phalloidin (magenta) and AmBNSab (green). (**A–H**) Images are shown at two different magnifications with an overview of the mushroom bodies (MBs) (top row) and details of KC nuclei at higher magnification (bottom row). Labeling with AmBNSab was absent at early larval stages (**A**,**E**), late larval stages (**B**,**F**)**.** At pupal stage 5, during the peak of postembryonic brain metamorphosis, only weak and sporadic labeling is visible in the cytosol of KCs (**C**,**G**). Labeling with AmBNSab in KCs of day 1 adult workers revealed typical rod-like assemblies in KC nuclei, although more sporadic and with shorter rod lengths compared to adult foragers (**D**,**H**). Scale bars in **A** (also for **B**) and **C** (also for **D**) = 50 µm, in **E** (also for **F**–**H**) = 10 µm. Number of brains analysed: foragers = 2, early larvae = 5, late larvae = 5, stage 5 pupae = 5, 1-day old adults = 4.

**Figure 5:**
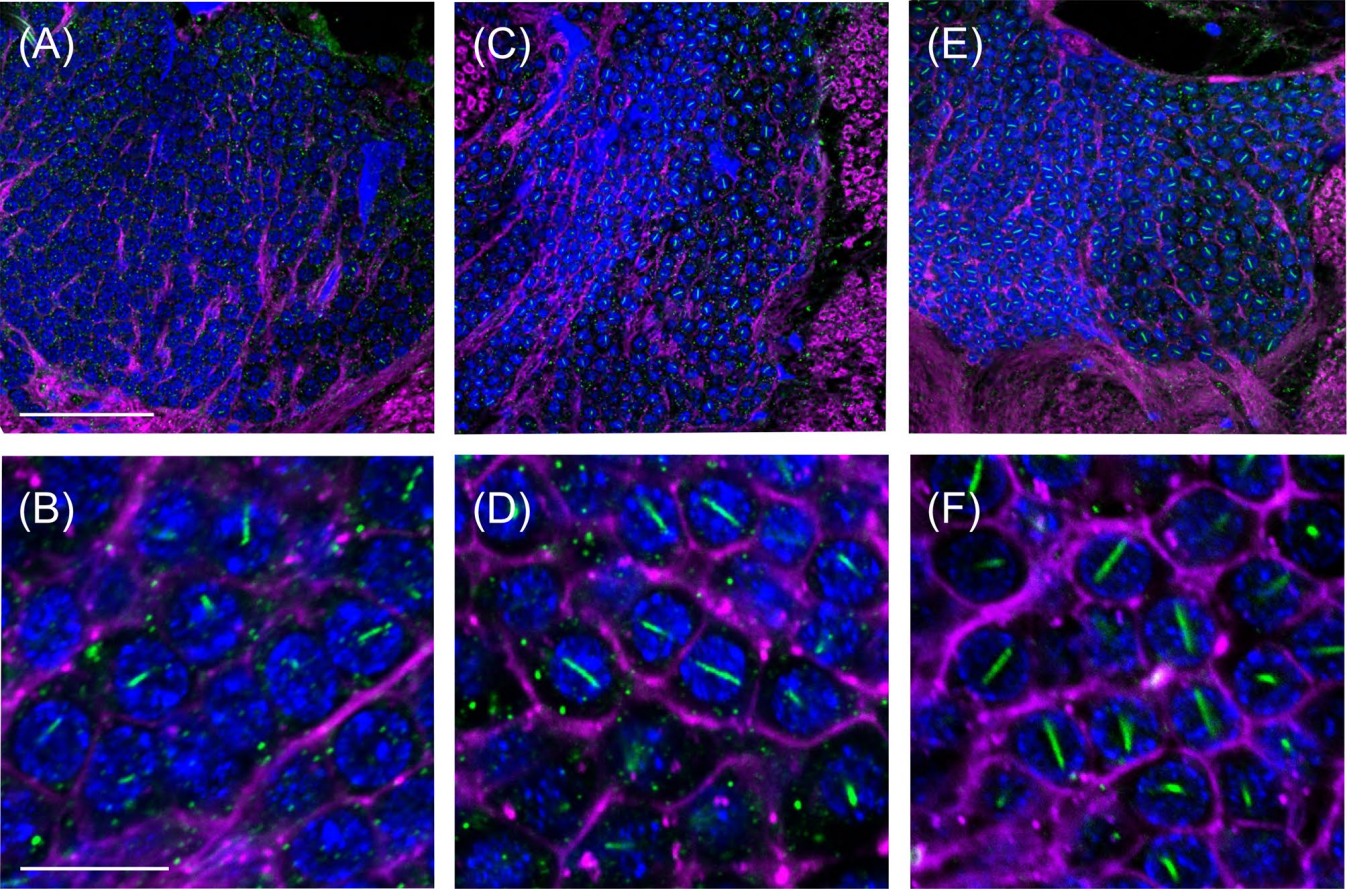
Caste- and sex-specific differences in labeling with AmBNSab. Staining with Hoechst 34580 (blue), f-actin phalloidin (magenta), and AmBNSab (green). (**A,B**) Example in a young (1-day old) virgin queen shows relatively short, rod-like assemblies in Kenyon cell (KC) nuclei labeled with AmBNSab compared to experienced female workers (foragers, **E**–**F**). (**C–D**) In the brain of a male (drone) rod-like assemblies in KC nuclei labeled with AmBNSab are comparable to those found in adult female foragers (**E**–**F**). (**E–F**) Positive control in an adult (female) worker bee (forager) shows typical rod-like assemblies of AmBNSab within different orientation in individual KC nuclei. Scale bars upper row (in **A**) = 50 µm, lower row (in **B**) 10 µm. Number of brains analysed: foragers = 10, young virgin queens, 2–4 days-old = 3, drones (unknown age) = 3.

**Figure 6:**
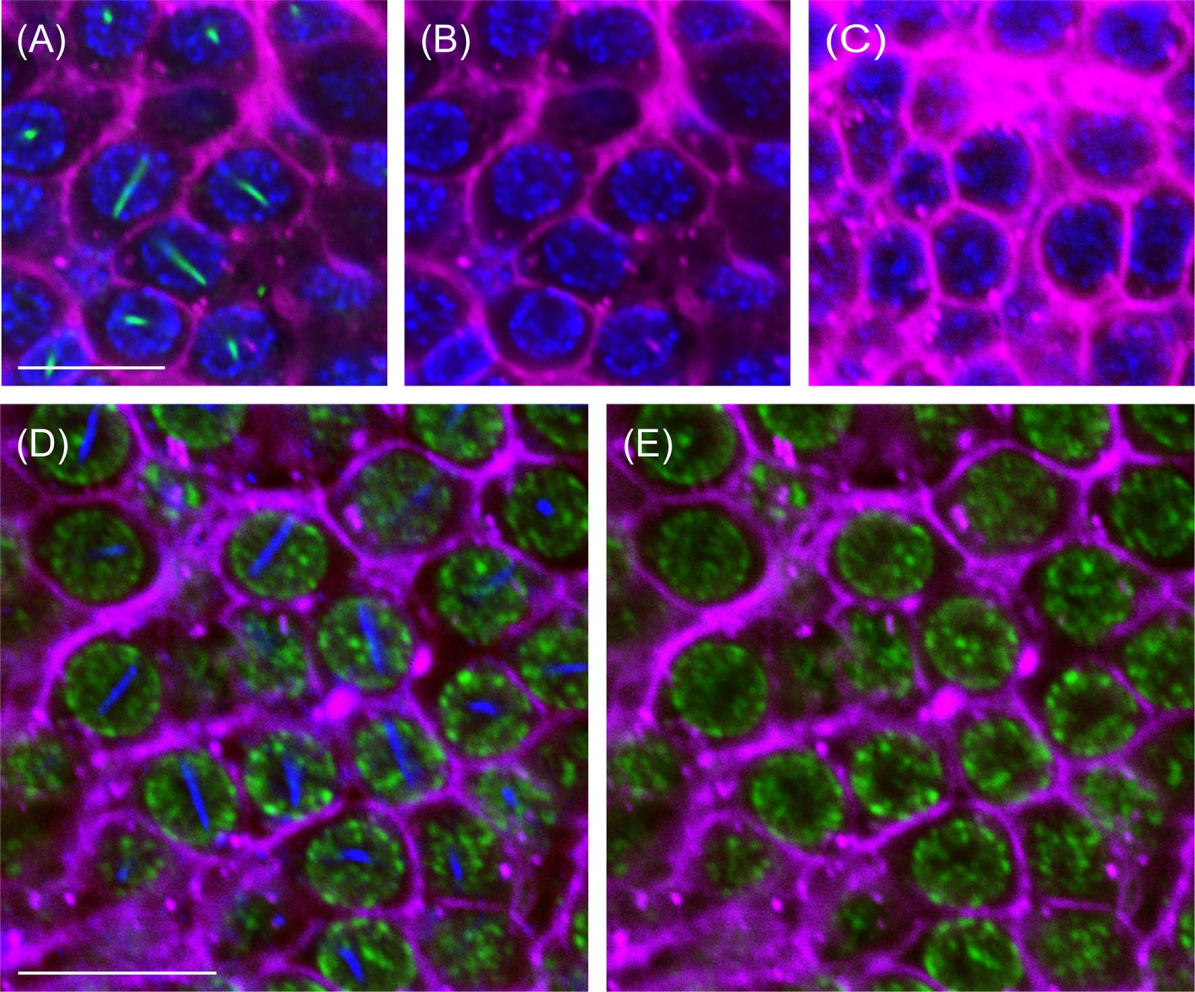
High magnification analyses of Kenyon cells (KCs) double labeled with AmBNSab and f-actin phalloidin in the brain of honey bee workers. (**A–C**) Labeling of KC nuclei with Hoechst 34580 (blue), f-actin phalloidin (magenta) and AmBNSab (green). Triple labeling in (**A**) shows that rod-like assemblies of AmBNSab do not colocalize with f-actin phalloidin. F-actin phalloidin labeled structures (**B**) do not reveal any structure related to the AmBNSab positive rod-like assemblies in (**A**). Strong excitation of the f-actin phalloidin (magenta) (**C**) suggests that the rod-like assemblies of AmBNSab (**A**) do not have any obvious association with the f-actin cytoskeleton inside the cell nucleus. (**D–E**) Triple labeling of KCs with Sytox green (green), f-actin phalloidin (magenta) and AmBNSab (blue). The rod-like assemblies of AmBNSab within Sytox-green labeled cell nuclei are clearly visible (**D**), and neither Sytox green, nor f-actin phalloidin labeling (**E**) shows any obvious association with the rod-like assemblies of AmBNSab labelling (**D**). Scale bar in (**A**) for upper row = 10 µm, in (**D**) for lower row = 10 µm. Number of forager brains analysed: with Hoechst 34580 = 3, with Sytox Green = 3.

Figure 1 shows an overview and the details of AmBNSab immunolocalization in the brain of adult honey bee workers. The overview reveals an evenly distributed light background fluorescence in all parts of brain (Fig. 1A). At high magnification, AmBNSab-labelled rod-like structures are clearly visible in nuclei of Kenyon cells (KCs), the intrinsic neurons of the MB a large proportion of which are housed within the cup shaped MB calyx (Fig. 1B,D,E,G). This applies to the inner compact, inner non-compact (class I) and the group of outer (class II) KC nuclei (Fig. 1C,E). We estimate the rods to be 3.7–4.1 µm in length in workers and drones and ~ 2.1 µm in young queens. Figure 2 shows examples of 3D reconstructions of individual KC nuclei from the group of inner non-compact KCs. The average length of rod-like assemblies obtained from randomly chosen KCs within individual worker bees (foragers, n = 10 in each case) was 3.7 µm for the class I inner compact KCs, 4.1 µm in the class I inner non-compact KC, and 3.6 µm in the class II outer KCs, which roughly correlates with the different size of the KCs in the three groups (for the spatial arrangement and characteristics of different KC groups, see Fig. 1 in Groh and Rössler^29^. The rods are positioned across the middle, but do not seem to be attached to the nuclear envelope (Figs. 1H, 2). Labelling is mostly absent or negligible in cell nuclei of other brain regions. For example, only a few neurons in the antennal lobes show labelling with AmBNSab (Fig. 1F,I) with virtually no signal detectable in other parts of the brain. To determine if AmBNSab detects a similar structure in other insects, we analysed brains of the fly *Drosophila melanogaster* and the carpenter ant, *Camponotus floridanus*. We were interested to examine if this novel structure is present in other insects, or perhaps is restricted to Hymenoptera, or even to one family of Apidae. As shown in Fig. 3, AmBNSab labelling is found in KCs of the honey bee with no binding detectable in KCs of both the fly and ant. To determine if the expression of this unusual nuclear structure is temporally regulated, we examined the AmBNSab labelling pattern in the larval and pupal stages. The results shown in Fig. 4 demonstrate that no labelling is detectable in both early (Fig. 4A,E) and late larvae (Fig. 4B,F). Interestingly, during pupal metamorphosis and a critical phase of brain development at pupal stage 5, only weak and sporadic labelling is visible around the cell nucleus (Fig. 4C,G). In contrast, in brains of young 1-day old workers, the structures with a typical rod-like shape are already visible, although they are shorter and not as frequent as in older brains (Fig. 4D,H). Although the labelling is well-defined in both female castes and drones, the rods are shorter in young queens (Fig. 5A,B) and more comparable to very young workers (Fig. 4D,H) than to adult workers or drones (Fig. 5C,D,E,F). In drones the rod-like assemblies were on average 3.6 µm long in the compact inner KCs, and 4.0 µm in the non-compact inner KCs, whereas in young virgin queens the average length in the compact and non-compact inner KCs was 2.1 µm (n = 10 in each case; in young queens, the variability was greater in comparison with foragers). To determine if the AmBNSab binding pattern colocalizes with the cytoskeletal protein f-actin, we used a combined AmBNSab and f-actin with phalloidin labelling (Fig. 6A,D). The choice of f-actin was motivated by recent evidence that in addition to its cytoplasmic function, this protein also has a nuclear role in organizing chromatin during mitosis. The labelling patterns shown in Fig. 6 rule out any spatial association between the rod-like structures and f-actin in the KC nuclei. To test for possible interferences between secondary antibodies and chromatin markers, two different staining combinations for cell nuclei (Hoechst 34580 and Sytox green) were combined with AmBNSab labelling. Both patterns reveal a very similar result regarding the rod-like assemblies (Fig. 6A,D). In addition, no related structures were detected in the chromatin labeling (for more details on all images see Figs. 1, 2, 3, 4, 5, 6 legends).

Taken together, our immunolocalization images show a unique and distinctly arranged protein assembly predominantly in intrinsic neurons of the MBs in adult honey bee brains. The intensity of immunostaining during postembryonic development is variable with no or very little labelling detectable during larval and pupal stages, and strong labelling seen in adult brains of worker bees, especially foragers, and drones, but weaker in young queens and 1-day-old workers. Such context-dependent expression is consistent with a potential biological role of this novel structure in the mushroom bodies (MBs), an important neuropil implicated in multimodal sensory processing, integration, associative learning, and memory formation^23,30–32^. One important feature of the MB neuropil is its volumetric plasticity in the absence of neurogenesis^13–15,33–37^. These volumetric changes and structural plasticity of synaptic complexes in the MBs occur during adult maturation of honey bees and are driven by both age and experience. In this context, our finding that this novel structure becomes more pronounced in older foraging bees suggests a possible connection to MB plasticity, and previously unexplored aspects of 3D architecture in the nuclei of Kenyon cells. Since the brain cells are postmitotic in adult honey bees^34^, the rod-like structure is mostly built after emergence in non-dividing KCs. It is noteworthy that cell cycle proteins are active in non-dividing neurons and their role in synaptic plasticity has been highlighted in several studies^38^. Axonal elongation, dendrite morphogenesis and synaptic maturation are all part of synaptic plasticity. While at this stage the connection between the novel nuclear structure and KC plasticity is purely speculative, the postmitotic dynamics of these neurons offers a promising avenue for further investigations.

While the lack of cross-species labelling is interesting, it is not unusual because lineage-specific gene inventions are quite common amongst insects^39–44^. Indeed, predicted proteins with no relatives in other species represent a functional void that can only be filled by multilevel analyses including antibody research. Alternatively, a related protein is present in these insects, but with sufficiently altered sequence preventing AmBNSab binding. At this stage, the target protein detectable by AmBNSab remains in the category of uncategorised, possibly honeybee-specific proteins, that needs to be further characterised. Moving from a cellular description of where and when this protein is found to biological function will need to incorporate a far greater component of cutting-edge biochemistry, including immunoprecipitation, peptide sequencing and cell sorting. We are confident that this goal can be promptly achieved, and our antibody will soon become a valuable tool in the study of brain and behaviour.

## Conclusion

We uncovered an intriguing nuclear structure in the Kenyon cells of the honey bee brain. Although the significance of this structure remains to be established, its context-dependent expression is suggestive of a role in nuclear architecture and possibly, the adult brain volumetric plasticity. This unexpected finding clearly shows that previously unexplored 3-D features of neurons can be visualised with powerful molecular tools including highly specific antibodies. An important aspect of this study is our demonstration that the nucleus still holds surprises that can be revealed with advanced bioimaging.

## Materials and methods

### Antibody generation

Initially we aimed to produce antibodies against the honey bee relative of mammalian TETs (Ten-eleven translocation methylcytosine dioxygenases) by targeting highly conserved catalytic regions of the predicted honey bee TET splice variants^24^ with the construct used to obtain the crystal structure of human TET2 (PDB: 4NM6)^45^ using MUSCLE (MUltiple Sequence Comparison by Log-Expectation). This alignment was used to generate a cropped consensus splice variant encompassing the structurally characterised catalytic domain of AmTET^24^. This sequence was analysed using peptide immunogenicity prediction software at Eurogentec (Liege, Belgium). Two of the suggested peptides were selected (CLRRSGLEEKILTIVK and CVVTMTKHRTLSKPED, C-terminally amidated) for synthesis and further immunisation at Eurogentec. In total, two rabbits were immunised with a combination of both peptides using a 28-day protocol. Rabbit immunisation was done under the strict rules of Eurogentec that complies with the highest standards for animal welfare, including the Federation of European Laboratory Animal Science Associations (FELASA), the Belgian Accreditation Body (BELAC), and the UK Home Office Animals Scientific Procedures Act. Ethical approval of the work was obtained from the EU Animal Experimentation Ethics Committee (Protocol Number A2016/12).

### Western blotting

Adult worker brains were homogenised in PBS containing cOmplete protease inhibitor cocktail (Roche) using a plastic pestle. An equal volume of SDS-PAGE loading buffer was then added, and samples were heated to 95 °C and incubated for 5 min. Samples were then cleared by a 5 min, full-speed centrifugation in a microcentrifuge. Cleared lysates were loaded onto a 6% SDS-PAGE gel and resolved until the dye migrated out of the gel. Afterwards, the resolved gel was blotted onto a Protran nitrocellulose membrane (GE Healthcare). Transfer was carried out for 16 h at 30 V / 90 mA in CAPS transfer buffer (10 mM Na-CAPS, 10% methanol; pH 11) using a Mini Trans-Blot chamber (Bio-Rad). Membranes were blocked with 5% skim milk dissolved in PBST for 1 h at RT. Immune sera were diluted 1:500 in the blocking solution and incubated with the membrane overnight at 4 °C. The membrane was then washed three times for 10 min with PBST. Anti-rabbit IgG HRP conjugated secondary antibody (#31,462, Thermo Fisher) was diluted 1:30,000 in blocking solution and incubated with the membrane for 1 h at 4 °C and washed three times for 10 min with PBST. Blot was developed using Pierce ECL substrate (ThermoFisher) and visualised with a Biorad ChemiDoc Touch Imaging System.

### Antibody validation with heterologously expressed HA-tagged Tet proteins

Cloning of the HA-tagged catalytic domains of *A. mellifera* and *H. sapiens* Tet proteins into a pcDNA3 mammalian overexpression vector has been described previously^24^. To determine if the new antibodies recognised AmTet, HEK293 cells were grown to 50–60% confluency in a 6-well plate and transfected with 1.5 µg of plasmid DNA per well, using a standard PEI protocol. After 24 h, cells were washed twice with PBS and harvested. Collected cells were suspended in SDS-PAGE loading buffer containing cOmplete protease inhibitor cocktail (Roche), and heat-lysed at 95 °C for 5 min. Lysates were cleared by centrifugation and loaded onto an 8% SDS-PAGE gel. Subsequent western blotting was carried out as described earlier.

### Animals

Different life stages of honey bee (*Apis mellifera carnica*) workers, young virgin queens, and drones were obtained from colonies reared at the institutional (Zoology II) apiary at the Biocenter, University of Würzburg, Germany. Staging of larvae and pupae was done according to Groh and Rössler^27^. For all neuroanatomical procedures, bees were anaesthetized on ice and then mounted for preparation.

### Immunohistochemistry

This protocol is described in more detail in our previous work^27,35^. For brain dissections, heads, or the entire bodies in the case of larvae and pupae, were covered with physiological saline (130 mM NaCl, 5 mM KCl, 4 mM MgCl_2_, 5 mM CaCl_2_, 15 mM Hepes, 25 mM glucose, 160 mM sucrose; pH 7.2). A window was cut in the head, and glands, tracheae and the pharynx were removed. Brains were immediately transferred into ice-cold 4% formaldehyde (methanol free, 28,908, Fischer Scientific, Schwerte, Germany) in phosphate-buffered saline (PBS; pH 7.2) and fixed at 4 °C overnight. Fixed brains were washed with PBS, embedded in 5% low-melting point Agarose (Agarose II, no. 210–815, Amresco, Solon, OH) and sectioned in frontal planes at 80–100 µm using a vibrating microtome (Leica VT 1000S, Nussloch, Germany). Sections were washed in PBS with 2% Triton-X 100 (1 × 10 min), PBS with 0.2% Triton-X 100 (2 × 10 min) and pre-incubated in PBS with 0.2% Triton-X 100 and 2% normal goat serum (NGS, DIANOVA GmbH, Hamburg, Germany) for 1 h at room temperature on a shaker. Afterwards, sections were incubated in AmBNSab antibody (rabbit) (1:1000) in PBS with 0.2% Triton-X 100 and 2% NGS for 2 days at 4 °C. Then, sections were incubated in secondary antibody (1:250, Alexa Fluor 488 and 405, goat anti rabbit, Kat.No. A-11008 and A-31556, Invitrogen by Thermo Fisher Scientific, Carlsbad, CA, USA) in PBS with 1% NGS. To label f-actin, CF633 Fluor conjugated Phalloidin (0.2 units, Kat.-Nr. 00,046, Biotium, Hayward, CA, USA) was added, incubated over night at 4 °C and then rinsed in PBS (2 × 10 min). For cell nuclei labelling, preparations were incubated in Hoechst 34580 (1:1000, Kat.No. H21486, LifeTechnologies GmbH, Darmstadt, Germany), or, in some cases, in Sytox Green (1:1000, Kat.No. S7020; LifeTechnologies GmbH, Darmstadt, Germany) in PBS for 15 min at room temperature, and then rinsed in PBS (4 × 10 min). Finally, sections were transferred in 60% Glycerol in PBS for 30 min and mounted on slides in 80% Gylcerol in PBS. Cover slips were sealed with nail polish and preparations kept in the dark at 4 °C until microscopy or − 20 °C for long-term storage.

### Laser scanning confocal microscopy and 3D-image processing

Brain samples were scanned with a confocal laser scanning microscope (Leica TCS SP2 and SP8, Leica Microsystems AG, Wetzlar, Germany) using a 20 × or 63 × water immersion objective (20.0 x /0.7 NA or 63x /1.20NA). Additional digital zoom was applied during image acquisition. Preparations were excited at wavelengths of 405 (for Hoechst 34580), 488 (for Sytox green and CF488), 568 (for A568) and 633 (for CF633) depending on the fluorophores used. All samples were scanned at a resolution of 1,024 × 1,024 pixels in xy-direction. For 3D image stacks, samples were scanned at z-step size of 0.5 µm. Single images or image stacks were processed using ImageJ (ImageJ 1.52p; Wayne Rasband, NIH, Bethesda, MD) and CorelDrawX9 (Version 21.0.0.593, Corel Corporation, Ottawa, OB, Canada). If necessary, contrast was adjusted in ImageJ or Corel Draw. Three-dimensional reconstructions of confocal image stacks were generated using the software Amira 2019.1 (FEI, Visualization Sciences Group; Hillsboro, OR; http://thermofisher.com/amira-avizo). The 3D surface models were generated using the SurfaceGen module.

## Supplementary Information

Supplementary Information
