# Supplementary material for: Novel structure in the nuclei of honey bee brain neurons revealed by immunostaining
Source: Sci Rep. 2021 Mar 25;11:6852. doi: 10.1038/s41598-021-86078-5 (PMC7994413; doi:10.1038/s41598-021-86078-5)

Supplementary figure

Figure S1

Characterisation of AmBNSab. **a.** Western blot of adult forager whole brain extract. AmBNSab recognised a protein of approximately 300 kDa. **b.** Western blots of HEK 293 cells transfected with HA-tagged *Apis mellifera* Tet catalytic domain ($AmTet_{cat}$; 150 kDa) or HA-tagged human Tet catalytic domain ($HumTet_{cat}$; 100 kDa). AmBNSab failed to detect either HA-tagged Tet protein. Both panels are faithful representation of the original gels with no digital enhancement used. The images show everything that was visible on each gel.

Figure S1.

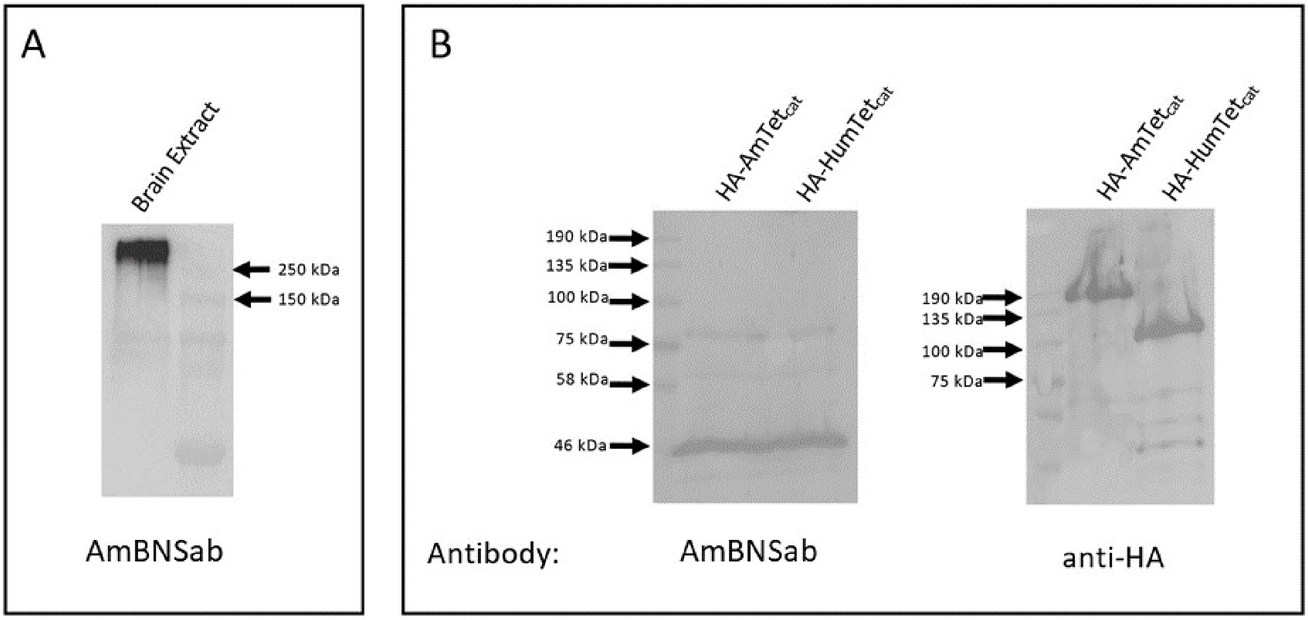

Supplement: Supplementary file 1 — Supplementary Information [file 41598_2021_86078_MOESM1_ESM.pdf]
